# Epsilon-caprolactone-modified polyethylenimine as a genetic vehicle for stem cell-based bispecific antibody and exosome synergistic therapy

**DOI:** 10.1093/rb/rbac090

**Published:** 2022-11-02

**Authors:** Yan Tan, Jiali Cai, Zhiyong Wang

**Affiliations:** Guangdong Key Laboratory for Biomedical Measurements and Ultrasound Imaging, School of Biomedical Engineering, Health Science Center, Shenzhen University, Shenzhen 518060, China; Key Laboratory for Polymeric Composite and Functional Materials of Ministry of Education, School of Materials Science and Engineering, Center for Functional Biomaterials, Sun Yat-Sen University, Guangzhou 510275, China; Key Laboratory for Polymeric Composite and Functional Materials of Ministry of Education, School of Materials Science and Engineering, Center for Functional Biomaterials, Sun Yat-Sen University, Guangzhou 510275, China

**Keywords:** polyethylenimine, stem cell, bispecific antibody, exosome

## Abstract

Bispecific antibodies (BsAb) have gained significant momentum in clinical application. However, the rapid enzymolysis and metabolism of protein drugs usually induce short circulation *in vivo*, and developing an efficient protein delivery system still is a bottleneck. Mesenchymal stem cells (MSCs) have become an attractive therapeutic carrier for cancers. Genetic modification enables MSCs to express and secrete specific proteins, which is essential for therapeutic efficacy. However, efficient gene transfer into MSCs is still a challenge. In this study, we applied epsilon-caprolactone-modified polyethylenimine (PEI-CL) as an efficacy carrier for plasmid transfection into MSC that served as *in situ* ‘cell factory’ for anti-CD3/CD20 BsAb preparation. Herein, the PEI-CL encapsulates the minicircle plasmid and mediates cell transfection efficiently. Thus, the anti-CD3/CD20 BsAb is secreted from MSC and recruited T cell, resulting in highly sensitive cytotoxicity in the human B-cell lymphoma. Furthermore, these stem cells produce exosomes bearing MiR-15a/MiR-16, which could negatively regulate cancer’s oncogenes BCL-2 for adjuvant therapy. Meanwhile, high immunologic factors like tumor necrosis factor-α and interferon-γ are generated and enhance immunotherapy efficacy. The engineered MSCs are demonstrated as an efficient route for BsAb production, and these bioactive components contribute to synergistic therapy, which would be an innovative treatment.

## Graphical Abstract

**Figure:**
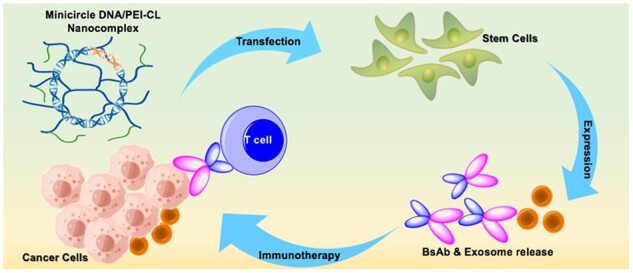

## Introduction

The bispecific antibody (BsAb) is designed with target specificities from two antibodies and it could connect with surface receptors on the T cells and tumor cells to bring them close as a linker and then trigger immunotherapy [1–3]. However, the traditional production of BsAb relied on the knobs-into-holes technology, which is based on the engineered bacterium host, the cumbersome expressions and complicated purification procedures [4]. By contrast, the mammalian host system could achieve post-translational medication that is required for the biological activity of BsAb [5]. Generally, the protein drug has rapid enzymolysis and metabolism [6], leading to lower therapeutic effect and becoming a significant drawback in clinical use [7]. Therefore, building a biological system to deliver antibodies effectively *in vivo* may be a perfect solution.

The excellent biocompatible, specific tumor-oriented migration and incorporation properties make mesenchymal stem cells (MSCs) an ideal targeted delivery vehicle [8–10]. Taking advantage of gene editing technology, the MSC could be endowed with other properties such as tumor-tropic and migratory. In this circumstance, if the BsAb gene could be applied to transfect stem cells, it is expected that stem cells can produce and secrete antibodies. Thus, the efficiency delivery of BsAb may be possible. Furthermore, some substances such as exosomes derived from MSC are also found with bioactive performances [11–13]. These secreted exosomes encapsulate with many proteins and microRNAs (miRNA), which could perform cell–cell communication, mediate many aspects of the physiological and pathological microenvironment and even affect the function of the target cells [14–17].

**Scheme 1.**
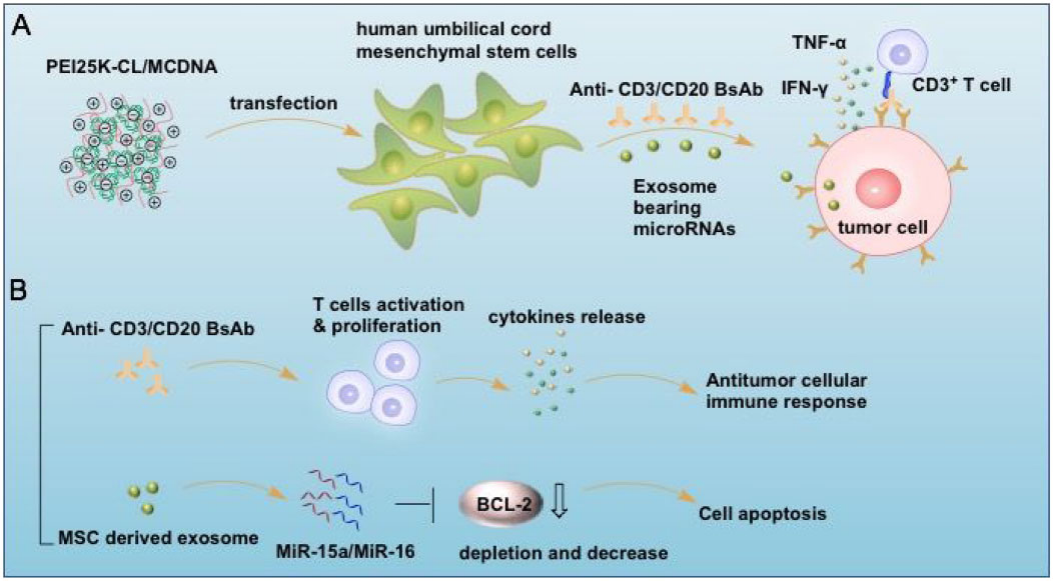
Engineered mesenchymal stem cells secrete bispecific antibodies and exosomes for B-lymphoma therapy. (**A**) Cationic PEI25K-CL carrier delivers MCDNA into the MSCs to produce anti-CD3/CD20 bispecific antibodies and exosomes. (**B**) The synergistic therapeutic effect of the antitumor immune response and cell apoptosis. MSC-derived exosome loading microRNAs of miR-15a/miR-16 could deplete BCL-2 expression and then induce the cell apoptosis; on the other hand, the released BsAb recruit activated T cells and perform antibody-dependent cellular cytotoxicity.

Herein, efficient gene delivery for MSCs is still a challenge. There are two categories of gene delivery systems: viral and non-viral. Unlike the viral system, non-viral vector such as a cationic carrier is safe and flexible for gene transfection [18]. In our previous work, epsilon-caprolactone-modified polyethylenimine (PEI-CL) was prepared as an efficient delivery vehicle for protein or nucleic acid owing to its excellent biocompatibility and high transfection efficiency [19, 20]. In this study, we used minicircle DNA plasmid as a versatile gene vector, which was devoid of bacterial plasmid backbone sequences providing excellent features in bio-safety and persistent transgene expression. And then, PEI-CL was applied to deliver the anti-CD3/CD20 BsAb expressed MCDNA into the human umbilical cord MSCs (HucMSCs). As a result, these genetically engineered cells become a metabolic manufactory to express anti-CD3/CD20 BsAb. Also, we extract exosomes secreted from MSCs, and find these exosomes carrying miRNAs, which could target and downregulate the anti-apoptosis gene. Finally, we proved that these antibodies and exosomes could activate T cells to inhibit B-cell lymphoma and perform an effective drug. Therefore, this study provides a new strategy for BsAb and exosome-based cancer therapy (Scheme 1).

## Materials and methods

### Main materials

Recombinant human interleukin-2 was from Minneapolis. The anti-Flag tag antibody was from Abcam. His tag enzyme-linked immunosorbent assay (ELISA) detection kit (No. L00440W) was purchased from GenScript. Anti-BCL-2 antibody was purchased from Abcam. The exosome extract kit (Exoquick-TC kit) was from SBI. Calcein-AM and PI were purchased from Life Technology. The Cytotox 96^®^ non-radioactive cytotoxicity assay kit was from Promega (Madison, USA).

### Cell lines and culture methods

HucMSCs were cultured as previously described [21]. Luciferase stable expression Raji cell line and the T cells were from the Chen-He Lab [22, 23]. T cells were generated from human peripheral blood mononuclear cells (PBMCs). To expand the cells, PBMCs were cultured in RPMI-1640 medium at a density of 1 × 10^6^ cells/well on a 24-well plate containing 2 mM of L-glutamine, 10% FBS and 100 U/mL of recombinant human interleukin-2.

### Atomic force microscope imaging

The PEI25K-CL/MCDNA complex (N/*P* = 20) sample (2 μL) was deposited on mica for 5 min. Then the sample was scanned by a Pico-Plus atomic force microscopy (AFM) with a tapping mode. The AFM characterization of the exosome was also measured.

### DLS experiments

The PEI25K-CL/MCDNA complex with different N/P ratios (0, 10, 20, 30) was prepared. The size and zeta potential were separately measured by a Malvern instrument.

### Anti-CD3/CD20 MCDNA transfection

Anti-CD3/CD20 mini-circle plasmid and HucMSCs were from the Chen-He lab [23]; 2 × 10^6^ HucMSCs were cultured in a 100-mm dish for 24 h. The appropriate concentration of PEI25K-CL encapsulated with 24 μg DNA (N/*P* = 20:1) in Opti-MEM for 30 min and formed nucleic acid nanoparticles. Then the nanoparticle complexes were added to the HucMSCs culture medium. After 6 h incubation, medium was replaced by the DMEM/F12 with 10% FBS, and the supernatant was collected for further analysis.

### Assess of anti-CD3/CD20 BsAb

Western blotting is used for qualitative analysis of the BsAb. Meanwhile, the semi-quantitation of BsAb is analyzed by indirect competitive ELISA. The GenScript His Tag ELISA Detection Kit was used according to the manual. The His tag antibody-coated plate was added with samples and standards, then Anti‐His Monoclonal Antibody was also added and incubated at room temperature for about 0.5 h. After washing the plate with washing solution four times, the Antibody Tracer was added. At last, the plate is read at 450 nm immediately. The amount of His‐tagged protein in the sample is determined by extrapolating its OD value to the standard curve. All standards and samples are prepared in duplicate.

### Luciferase quantitative and lactate dehydrogenase analysis of the cytotoxicity

CIK and luciferase–Raji cells at an 8:1 ratio were mixed together in 96-well plates. The supernatant of the transfected HucMSCs (18 pg/μL BsAb) and exosomes (24 μg/5000 target cells, 24 μg/100 μL) were added and incubated for 6 h. The luciferase expression of the cells was evaluated using the Xenogen *in vivo* animal imaging system (IVIS)-100 system. Cytotox 96^®^ non-radioactive cytotoxicity assay (Promega, Madison, USA) was performed for the detection of lactate dehydrogenase (LDH) released analysis. After 6-h incubation at 37°C, 50 μL supernatant from each well was used in the LDH assay. OD490 was measured to calculate CIK-mediated specific cytotoxicity toward target cells. The percentage of specific lysis was calculated according to the following equation:

$$\%\text{specific lysis}=\frac{\text{experimental release-spontaneous release}}{\text{maximum release-spontaneous release}}\times 100\%$$

### T cell cytotoxicity toward Raji

Loading of target cells with calcein-AM as the specific protocol indicates. The calcein-AM is used at a final working concentration of 5 μM. After staining at 37°C for 15 min, cells were washed in a dye-free buffer. Then, stained cells were incubated with T cells at an 8:1 E:T ratio, in the presence or absence of supernatant of the transfected HucMSCs, which contained 18 pg/μL BsAb. After 4 h, the supernatant was collected into a black side 96-well plate, and fluorescence was measured using a Microplate Reader (Tecan Infinite M1000 PRO) at 485 nm excitation/520 nm emission. Results were measured as percentage lysis with respect to cells lysed with 1% SDS (100% lysis) after background subtraction (spontaneous calcein release).

### Determination of cytokine concentration

CIK and Raji cells are mixed together in 96-well plates at an 8:1 ratio. The density of Raji cell is 10 000/well. The supernatant of the transfected HucMSCs (18 pg/μL BsAb) was added and incubated for 24 h. The ELISA Sets (Biolegend, USA) were used to determine the interferon (IFN-γ) and tumor necrosis factors (TNF-α). Experiments were performed in triplets.

### Exosome extraction

HucMSCs were cultured in a 75 cm^2^ flask for 24 h, and the density was ∼80–90%, the medium was replaced by DMEM/F_12_ without FBS. After 24 h or 48 h, the culture medium was collected and centrifuged at 1000 rpm for 5 min to remove the remaining cells. Next, the cell supernatant was condensed/concentrated by using Millipore ultrafiltration centrifuge tubes (MWCO =100 KDa) at 4000 g for 30 min to a volume of 200 μL. Then the Exoquick-TC kit was used to obtain the exosomes.

### Cellular uptake of MSC-derived exosomes

HucMSCs-derived exosomes were labeled with PKH67 (Sigma-Aldrich, St. Louis, MO) as previously described with minor modification. In brief, 2 μL of PKH67 was added to 25 μg of MSC-derived exosomes in a total of 1 mL of diluent and incubated for 20 min at room temperature. One milliliter of 1% bovine serum albumin was added to stop labeling and the mixture was added into 18 mL of phosphate buffered saline (PBS) and centrifuged at 100 000 g for 2 h at 4°C. The supernatant was removed and the pellet was resuspended in 20 mL of PBS and then centrifuged at 100 000 g for 2 h at 4°C. The pellet containing PKH67-labeled exosomes was resuspended in 2 mL of R1640. Raji cells were previously cultured and the medium was replaced with R1640 containing PKH67-labeled exosomes and cells were incubated for 24 h at 37°C with 5% CO_2_. After incubation, cells were washed twice with PBS and fixed in 4% paraformaldehyde for 20 min at room temperature. The sample was washed twice with PBS. The Hoechst 33258 was used to stain the cell nucleus. Cellular uptake of MSC-derived exosomes was observed under a confocal laser microscopy (TCS SP5II, Lexica, Ernst-Leitz-Strasse, Germany).

### RNA isolation and real-time quantitative PCR

Total RNA was isolated from exosomes with RiboPure™ RNA Purification Kit (Invitrogen™) according to the manufacturer’s instructions. miRNA quantitation was performed with Mir-X™ miRNA real-time quantitative PCR (qRT-PCR) SYBR^®^ Kit (Cat.638314, Clontech). The primers are as follows: miR15a: 5′-TAGCAGCACATAATGG-3′; miR16: 5′-GCGGCGGTAGCAGCACGTAAAT-3′, U6: 5′-ATCCAGTGCAGGGTCCGAGG-3′, the reverse primer is universal primer. Relative gene expression levels were calculated using ΔΔCT analysis by the following formula: ΔΔCT = ΔCT of Sample – ΔCT of Calibrator. Relative Gene Expression = 2^−(ΔΔCT)^.

### Statistical method and graphic

Data present as mean ± SD. Statistical analysis is performed by using SPSS 16.0 and GraphPad Prism 6.

## Results and discussion

### Cationic PEI25K-CL as an effective vehicle for minicircle plasmid transfection

We used our high-performance transfection reagent PEI25K-CL as a MCDNA vehicle in this study. The MCDNA plasmid (MC.CD20) was a gift from the Chen-He lab. The structure map of MC.CD20 is shown in Supplementary Fig. S1A. PEI25K-CL was prepared via the epsilon-caprolactone-based ring-opening reaction on the amine group from polyethylenimine (Supplementary Fig. S2A). Herein, the substitution was calculated at ∼12% from the nuclear magnetic resonance spectroscopy (NMR) data (Supplementary Fig. S2B).

**Figure 1.**
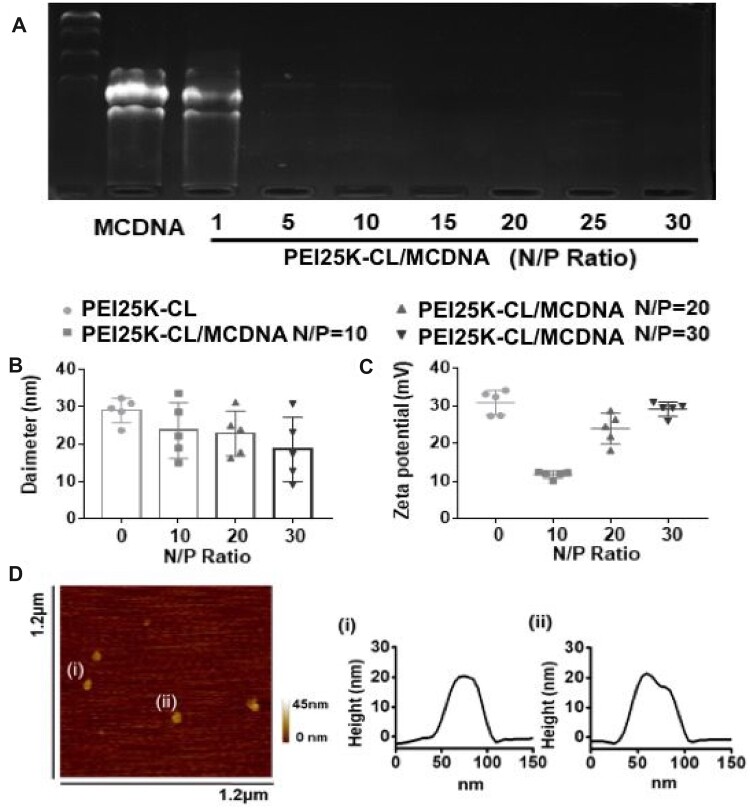
Characterization of PEI25K-CL/MCDNA genedelivery system. (**A**) The agarose gel electrophoresis analysis of free MCDNA and PEI25K-CL/MCDNA. (**B**) DLS results of PEI25K-CL/MCDNA. (**C**) Zeta potential of PEI25K-CL/MCDNA. (**D**) AFM height image and analysis of PEI25K-CL/MCDNA (N/P ratio is 20).

Furthermore, the gene loading capacity of PEI25K-CL was analyzed by gel electrophoresis in Fig. 1A. When the N/P ratio was higher than five, nearly all MCDNA molecules were trapped with less mobility, indicating that PEI25K-CL could efficiently encapsulate MCDNA. As known, different physicochemical properties of nanoparticle influence the cellular uptake efficiency. Therefore, DLS was used to test the size distribution and Zeta potential of PEI25K-CL/MCDNA nanocomplexes. Figure 1B and C shows that the size of blank PEI25K-CL is 29.04 nm (± 2.96 nm), and the Zeta potential is +30.87 mV (± 2.94 mV). When loaded with MCDNA, the nanocomplexes size was slightly decreased to form a more compact nanostructure around 20 nm during the interaction between PEI25K-CL macromolecule and nucleic acid. The zeta potential also decreased from +29.12 mV to +11.77 mV with the N/P ratio reducing from 30 to 10, which was attributed to the increased amount of negatively charged MCDNA. AFM was also applied to characterize the samples. As shown in Fig. 1D, PEI25K-CL/MCDNA complex (N/P ratio 20) was separately dispersed with a size of ∼20 nm, which was similar to the results of DLS. These results demonstrated that the MCDNA plasmid could load onto the PEI25K-CL and form a stable nucleic acid-loaded particle complex.

### Anti-CD3/CD20 BsAb expressed by HucMSCs

**Figure 2.**
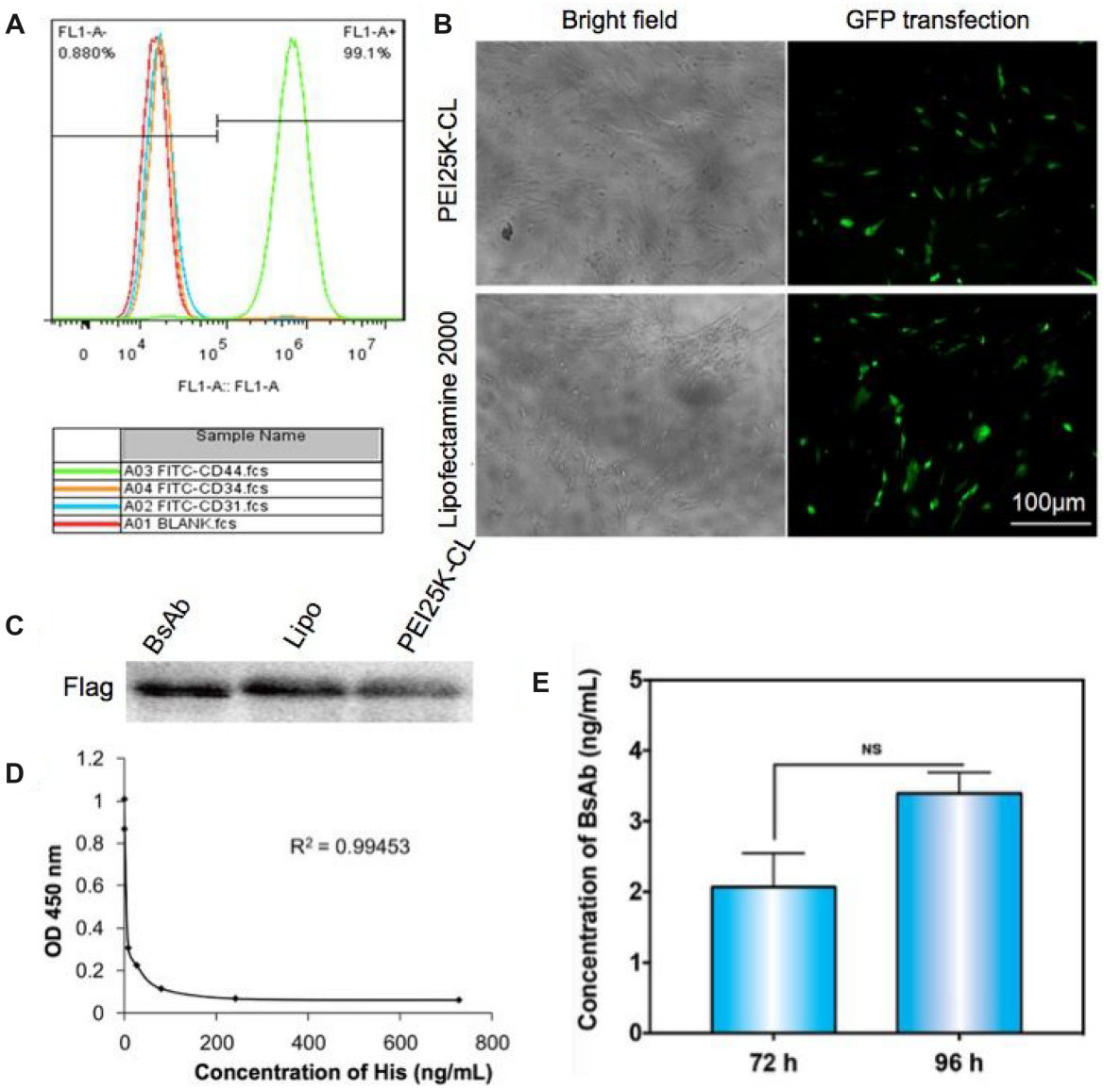
Transfection of HucMSCs by PEI25K-CL. (**A**) Characteristic of HucMSCs by flow cytometry. (**B**) PEI25K-CL and lipofectamine 2000 mediated the GFP MCDNA transfection in HucMSCs. (**C**) The BsAbs in the culture medium of PEI25K-CL transfected HucMSCs measured by WB, and the secreted BsAb was evaluated by anti-Flag antibody. Lipo represents lipofectamine 2000-mediated transfection system. (**D**) The standard curve of His concentration by ELISA. (**E**) BsAb concentration was determined after 72 and 96 h of transfection. T-test. *P *=* *0.0799. NS: no significance difference.

The MSCs were cultured and characterized. After staining with different fluorescently labeled antibodies, these cells showed highly expressed CD44; on the contrary, they had the negative expression of CD31 and CD34. The results were consistent with the previously reported findings and confirmed that these extracted HucMSCs were of high purity [24, 25] (Fig. 2A). To investigate the gene transfection efficiency of PEI25K-CL/MCDNA in MSCs, the green fluorescent protein-based plasmid was applied. The commercialized liposome, lipofectamine 2000, was also used as the positive control vehicle. In Fig. 2B, PEI25K-CL yielded a similar transfection efficiency compared to commercial lipoplexes. Owing to its higher production yield and better stability, the PEI25K-CL was applied for BsAb gene transfection.

The nanocomplex of PEI25K-CL encapsulating anti-CD3/CD20 MCDNA was prepared and cultured with MSCs. After transfection, the cell culture medium was collected and the secreted BsAb was evaluated. Herein, for improving the stabilization and properties, the anti-CD3/CD20 MCDNA bearing antibody was designed with a linker-hinge domain connecting the his-tagged anti-CD3 scFvs and the flag-tagged CD20 antibody. Thus, according to the western blot analysis in Fig. 2C, the bands of the flag from the gene-transfected cells were observed, indicating the BsAb anti-CD3/CD20 was successfully secreted in the PEI25K-CL/MCDNA-treated MSCs. Furthermore, the concentration of BsAb in the culture media was assessed using His tag ELISA detection kit. Based on the four-parameter equation fitting method, the nonlinear least square has been applied to estimate the parameters (Fig. 2D). Accordingly, the BsAb concentration is ∼18 ng/ml per 10^6^ cells. Moreover, the expression level of BsAb from stem cells has no difference after 72 and 96 h of transfection, indicating the BsAb secretion could be detected more than 4 days after transfection (Fig. 2E).

**Figure 3.**
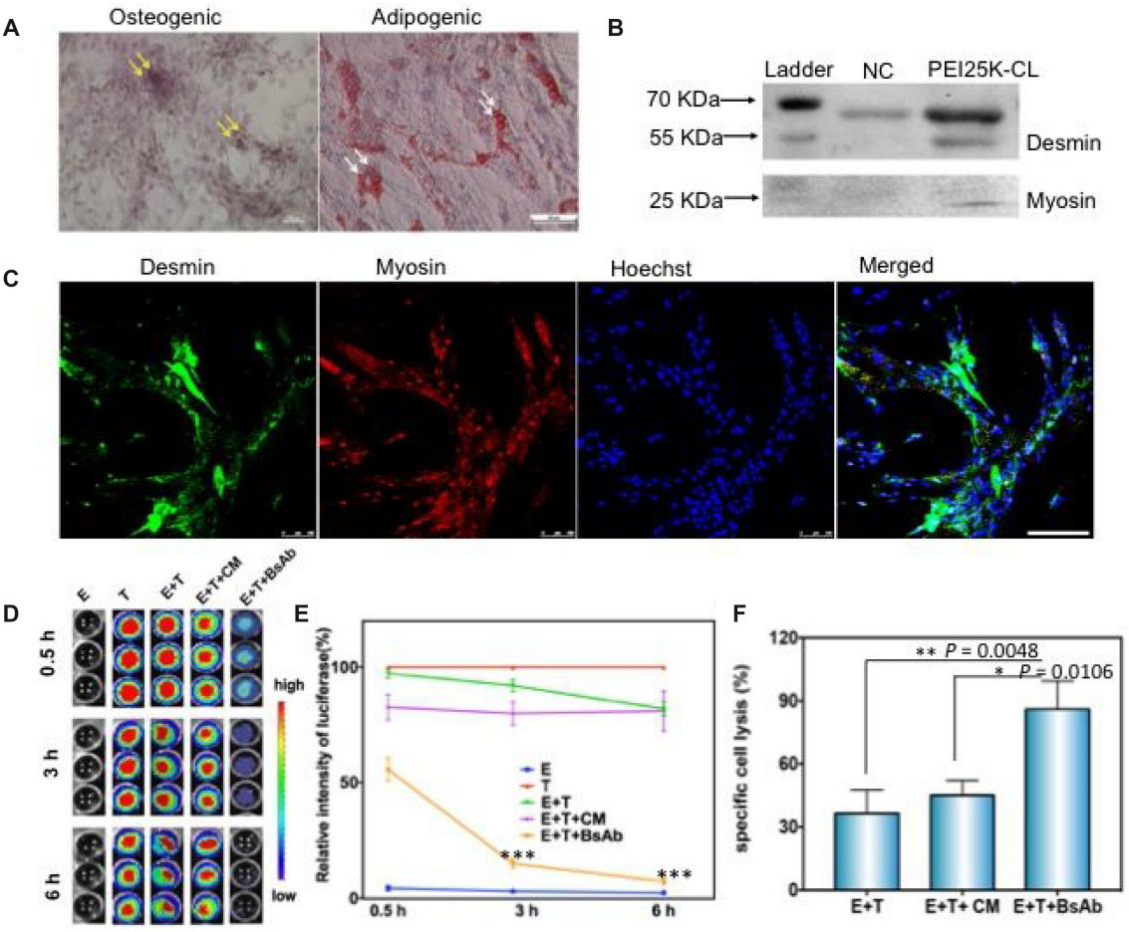
(**A**) Osteogenic, adipogenic differentiations measured by alizarin red and oil red O staining. (**B** and **C**) myogenic differentiation ability of the transfected HucMSCs evaluated by WB experiment and confocal imaging. The scale bar is 100 μm. NC is short of negative control, which is the sample from the normal HucMSCs cells. (**D**) IVIS imaging of Raji–luciferase cells after incubating with CM and BsAb. (**E**) Statistic analysis of the cell activity ratio according to the luciferase imaging at different time points (E + T+BsAb vs E + T+CM, ***P <* *0.0001 at 3 h and 6 h). (**F**) Specific cell lysis qualification of the Raji cells after LDH assay. E + T means the cell mixture solution of T cells (effector, E) and Raji cells (target, T). E + T+CM means cells added with HucMSC culture medium. E + T+BsAb represents BsAb incubated with E and T cells. (*n* = 3. Data show with mean ± SD. *P <* *0.05, **P <* *0.01).

Multi-differentiation, including osteogenic, adipogenic and myogenic differentiation, is an important characteristic of MSCs. Thus, multipotency abilities of the transfected MSCs were also assayed. In Fig. 3A, the alizarin Red S staining showed amaranthine in the PEI25K-CL/MCDNA transfected MSCs after 21 days of osteogenic induction (white arrow). Otherwise, significant red oil droplets were exhibited in the cells treated with Oil Red O staining (yellow arrow). Additionally, the differentiation marker of myoblasts like Desmin (55 KDa) and Myosin (20 KDa) was detected [26]. The western blot experiment and confocal imaging showed the myogenic differentiation of the treated MSCs (Fig. 3B and C). These results demonstrated that the PEI25K-CL/BsAb-MCDNA nanocomplex transfected MSCs still possessed multilineage differentiation potential and could act as a living cell factory for BsAb production.

### Antibody-dependent cellular cytotoxicity by anti-CD3/CD20 BsAb

In clinical application, the BsAb can induce antibody-dependent immunotherapy [27]. In our study, the biological activity of the secreted protein was measured. The 30 μl culture media containing anti-CD3/CD20 BsAb at a concentration of 18 pg/μl was added to the 96-well black-side plate along with the effector (cytokine-induced killer: CIK cells, E) and target cells (tumor cells, T). As known, in the presence of ATP and oxygen, luciferase could catalyze the substrate luciferin with the production of light. The Raji tumor cells expressing firefly luciferase were very convenient for us to evaluate the effect of apoptosis through an IVIS. As shown in Fig. 3D, luciferase intensity dropped remarkably as time extended in the BsAb added group (E + T+BsAb) and its tumor cell activity decreased significantly compared with the E + T group after treatment of 6 h (Fig. 3E). To further determine the natural killer cell-induced antibody-dependent cell-mediated cytotoxicity (ADCC), the Calcein-AM staining method was established for living cell counting, and another quantitative method based on the LDH from lysed cells was used to evaluate ADCC. These two methods showed less restrictive and highly sensitive data and the proportion of specific lysis was higher in the BsAb-induced group (E + T+BsAb) than the other groups in Fig. 3F.

Overall, the two analyses demonstrated that BsAb could significantly enhance CIK cellular cytotoxicity’s effect on the Raji cells (*P *<* *0.05). Interestingly, the MSCs culture medium also gives influences on Raji cell lysis. We speculate that the secretions, such as some anti-cancer cytokines from MSCs, would positively impact cell cytotoxicity [28]. MSCs have also been reported to secrete exosomes with high levels of miR-15a [29–31]. The transfected MSC culture medium may have efficiency for tumor therapy due to the extracellular exosomes. Thus, these cell secretions would act as a tumor-suppressive role and may be potential key mediators of therapeutic action that can be the tendency of future treatments.

**Figure 4.**
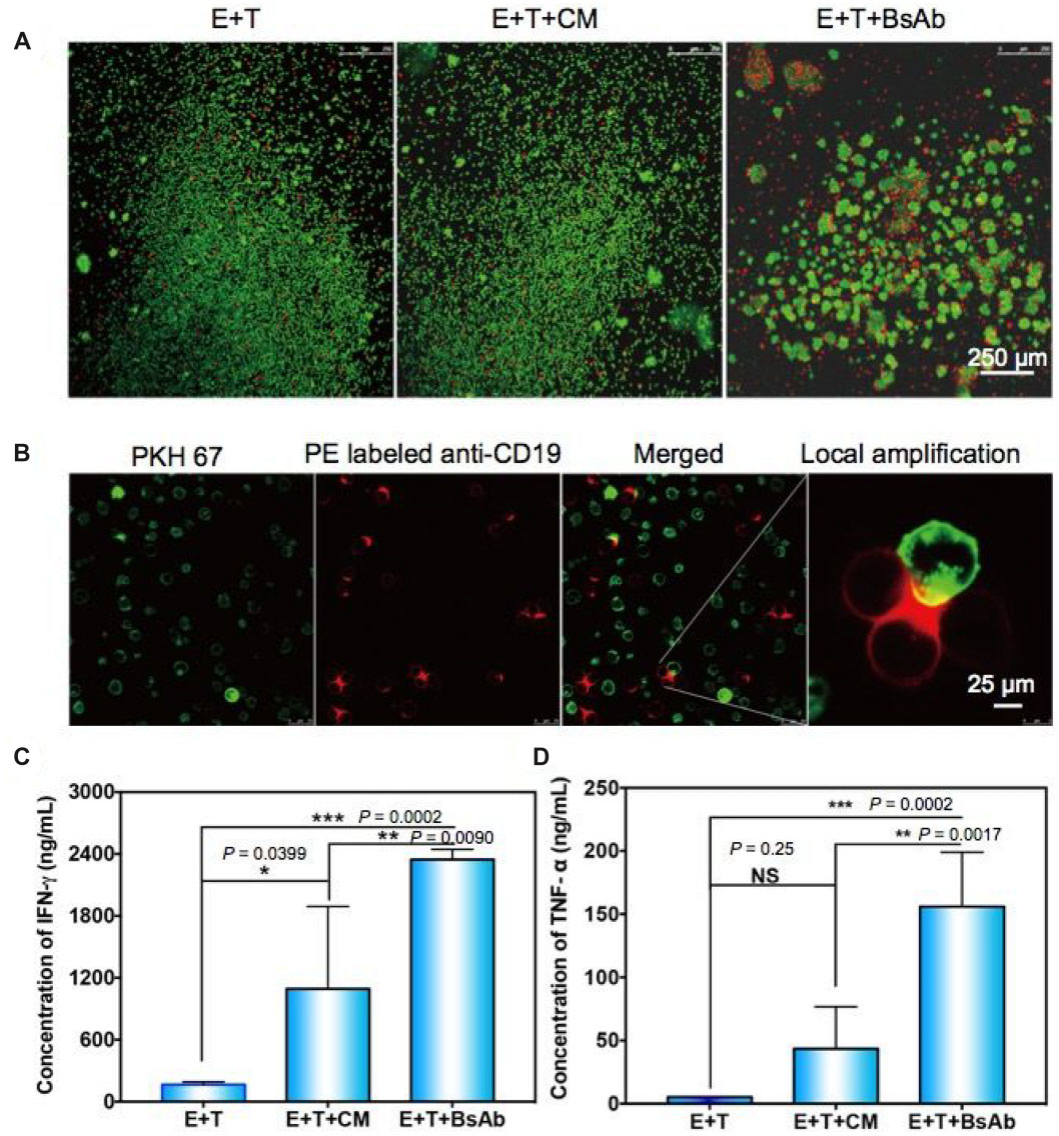
(**A**) Direct observation of the cytotoxicity by confocal imaging. Calcein-AM/PI staining. The live cells show green fluorescence, and the dead cells are red. (**B**) The interaction between CIK cells and Raji cells. PKH67 stained the CIK cells, while the anti-CD19 antibody labeled the Raji cells. (**C**) Statistic of the concentration of IFN-γ in the culture medium. (**D**) Statistic of the concentration of TNF-α in the culture medium. Data shown as mean ± SD, ***P <* *0.001; **P <* *0.01; *P <* *0.05. NS: no significant difference.

In parallel, the ADCC experiment was also measured by confocal imaging through the Calcein-AM and PI staining to detect the live and dead Raji cells. Significant cytotoxicity was clearly observed as the BsAb was added into the effector and target cells and co-cultured for 4 h in Fig. 4A. It showed that CIK cells were redirected and accumulated around tumor cells with an assistant directly from BsAb, leading to cancer apoptosis. Therefore, the number of PI-labeled cells (Red) was more than the control group. To further assess the bio-effect of BsAb, the CIK cells were stained with PKH67, and the Raji cells were labeled with anti-CD19 antibody PE. CD19 is another specific antigen expressed in Raji cells besides CD20 [32]. According to this method, we could directly observe the biological phenomena in Fig. 4B. Herein, the merged fluorescence (yellow) in the confocal imaging indicated a bio connection between the effector CIK cell and the target Raji cell. These results exhibited the secreted BsAbs possessed high bioactivity to trigger T cell-mediated cytotoxicity.

### Anti-CD3/CD20 BsAb activates CIK cells to kill Raji cells by releasing IFN-γ and TNF-α

As well known, the conjugation of the T-cell receptor with a major histocompatibility complex molecule could trigger the production of IFN-γ. And then, this IFN-γ activates macrophages, up-regulates many gene products and renders antitumor immunocytotoxicity [33, 34]. It is reported that the treatment with IFN-γ plus daratumumab^@^ significantly attenuated Acute Myeloid Leukemia tumor growth [29, 35]. Therefore, the IFN-γ becomes a major component responsible for the activated cytotoxic T lymphocytes mediated cancer immunoediting. In Fig. 4C, the expression levels of IFN-γ were significantly elevated in the (culture medium) CM containing anti-CD3/CD20 BsAb added group (*P *=* *0.0002), as the same tend compared with the negative control group that incubating with only MSC CM (*P *=* *0.0090). Interestingly, the IFN-γ in the E + T+CM-treated group has a significant expression level than in the E + T group (*P *=* *0.0399). Moreover, TNF-α, another crucial cytokine, was also activated in host immune responses and showed a significant difference between the E + T+BsAb group and other groups (Fig. 4D).

### Stem cells derived exosomes mediating Raji cell apoptosis

**Figure 5.**
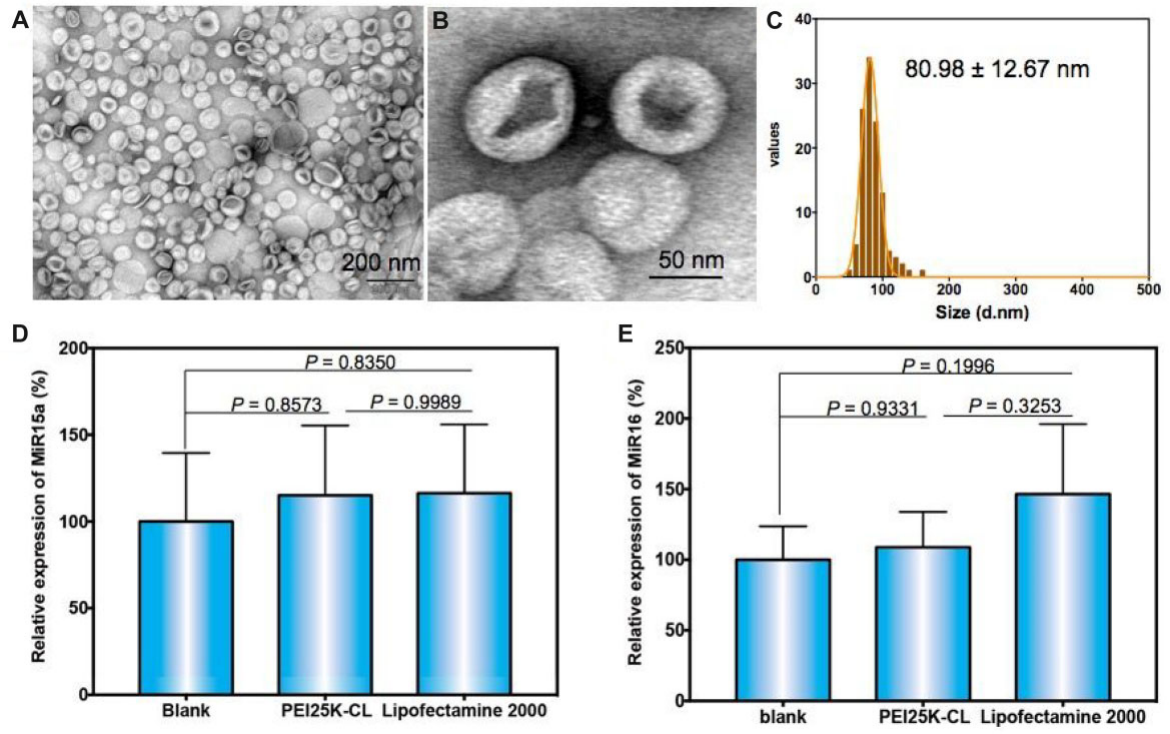
(**A**–**C**) Characterization of HucMSCs derived exosome: TEM and size distribution. (**D** and **E**) are the statistical data of the microRNA expression quantified by qPCR.

During our experiment, the culture medium from free MSCs exhibited a direct bioeffect against Raji cells. We speculate that the exosomes in the CM may play essential roles. Thus, we explored the cytotoxicity behavior and mechanism of exosomes from the MSCs. The exosomes were extracted, derived from the untransfected MSCs. The transmission electron microscopy image demonstrated that the isolated exosomes have a saucers-like shape with an average size of 81 nm in diameter (Fig. 5A–C), which was consistent with AFM results (Supplementary Fig. S3). Specific proteins of exosomes such as CD63, HSP70, CD9 and CD81 are subsequently assessed by western blot and the result was consistent with the expectation (Supplementary Fig. S4). Some evidence demonstrates that HucMSC-derived exosomes have various miRNAs such as miR-15a and miR-16 acted as tumor suppressors and protected against diseases, especially lymphocytic leukemia, through the downregulation of the target gene BCL-2 [16]. So the miR-15a and miR-16 in the exosomes could generate suppressive effects on the cancer cells [36]. Thus, we then checked the expression of MiR-15a and MiR-16 levels in the HucMSC-derived exosomes. As shown in the qPCR results (Fig. 5D and E and Supplementary Fig. S5), there are MiR-15a and MiR-16 expressed in the exosomes, and also the transfection with PEI25K-CL/MCDNA have no significant effect on the expression of MiR-15a and MiR-16.

The HucMSC-exosomes carried miRNAs and delivered them into the cytoplasm of the cancer cells. Hence, the cellular uptake of exosomes by Raji cells was verified via confocal imaging. In Supplementary Fig. S6, the exosomes membrane labeled with PKH67 appeared green and the cancer cell nucleus was stained by Hoechst 33258. The results showed that exosomes were effectively internalized by the Raji cell and located in the cytoplasm. In this case, HucMSC-derived exosomes served as an ideal carrier of MiR-15a and MiR-16 to the cancer cells.

**Figure 6.**
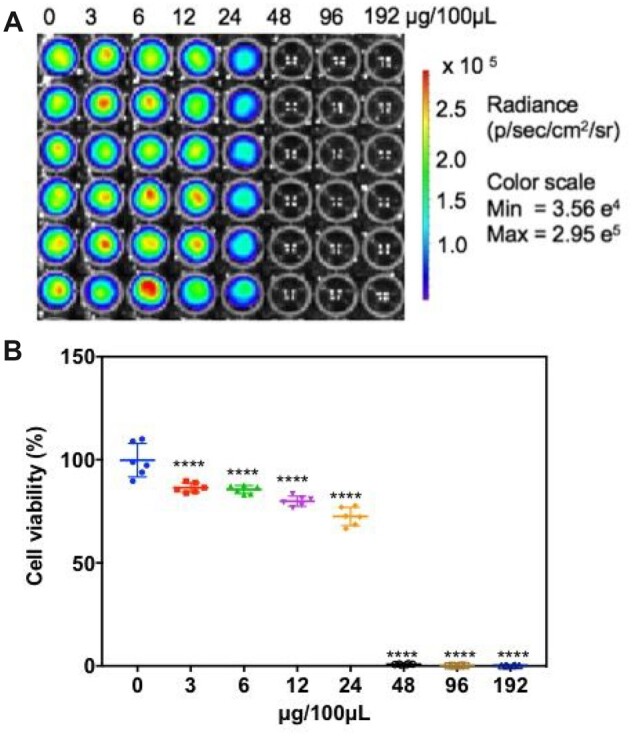
Cytotoxicity of the exosome to the target cell Raji. (**A**) IVIS imaging of the luciferase–Raji cells after incubating with different concentrations of exosomes. (**B**) Statistic of the cell viability. ****P <* *0.0001, compared with the 0 μg/100 μl treated group.

**Figure 7.**
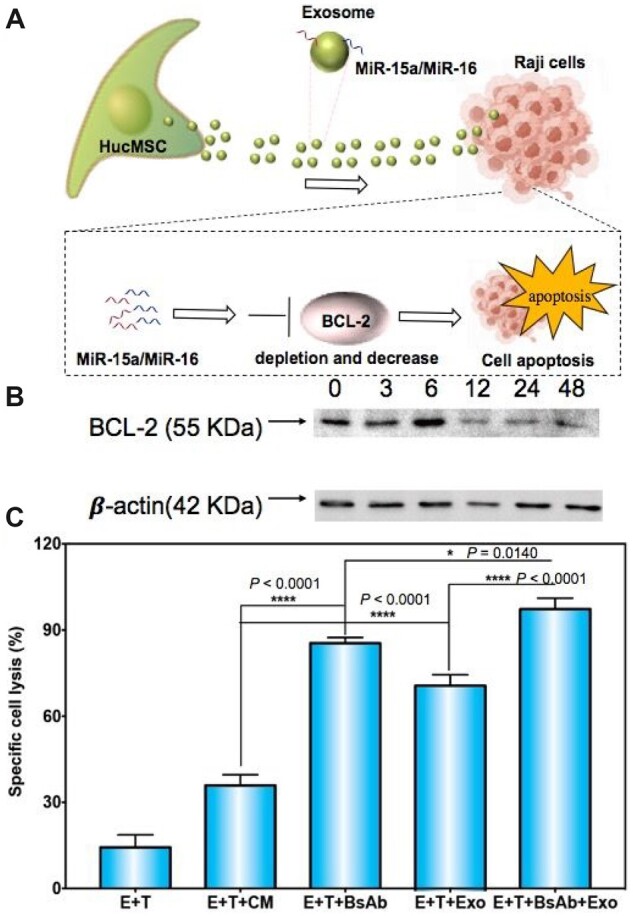
(**A**) The scheme of the exosome-mediated cell apoptosis. (**B**) BCL-2 protein expression after treatment of the exosomes at 0 – 48 μg/100 μL. (**C**) Cytotoxicity effect of BsAb and exosome on Raji cells. Exo: exosomes. Data presented as percent of cell death. *P <* *0.05, ****P <* *0.0001.

miRNAs on exosomes would transfer to tumor cells and inhibit the target gene [37]. To determine the cytotoxicity effect of the HucMSCs exosomes, luciferase-expressed Raji cells were treated with HucMSCs exosomes at gradient concentrations from 0 to 192 μg/100 μL. After 48-h incubation, the luciferase intensity was measured. As shown in Fig. 6A and B, the bioluminescence signal intensity was greatly suppressed as the exosome’s concentration increased (*n* = 6), which clearly indicated the cell viability of Raji declines significantly via the treatment with HucMSCs-derived exosomes. After that, the protein BCL-2 in Raji cells, which is the target gene of MiR15a and MiR16 (Fig. 7A), was assessed by western blot (Fig. 7B). The results confirmed that the expression of BCL-2 was downregulated by exosome-based therapy, and these exosomes mediated Raji cells apoptotic in a dose-dependent manner.

### Synergistic effect of BsAb and exosome on Raji cells

To further study the synergistic effect of BsAb and exosome on Raji cells, the ADCC experiment was performed with the 8:1 ratio of effector and target in a 96-well plate for 4 h of incubation, and the results were detected by LDH. As shown in Fig. 7C, compared with the E + T group, every treated group has significant cytotoxicity. E + T+BsAb-treated group mediated ADCC and induced close to 85% apoptosis of Raji cells. E + T+CM and E + T+Exo groups showed cell killing capacity with ∼35.86% and 71.00%, respectively. Interestingly, when the BsAbs and exosomes were mixed (E + T+BsAb+Exo group), the percentage of specific lysis was increased by close to 97.3% (contrast to the E + T+Exo group, *P *<* *0.0001; contrast to the E + T+BsAb group, *P *=* *0.0140). These preliminary results showed that HucMSC-derived exosomes carrying MiR15a and MiR16 have a positive suppression on Burkitt’s lymphoma cells. And this supplies a new strategy of synergistic therapy. On the other hand, this cell-free treatment may provide a new paradigm for MSC therapy [38]. The inhibition mechanism of exosomes and *in vivo* measurement are necessary for our further study.

## Conclusion

The application of BsAb is broad and fast-growing in immunotherapy against cancer and infectious diseases. Some of them have been currently applied in clinical testing. Nonetheless, there are still considerable challenges in the therapy. Transfected MSC as a production factory has satisfactorily optimized the expression of BsAb. Briefly, BsAb secreted from MSCs exerts a strong ADCC response on B cell malignancy at low concentrations and shows a new paradigm of the connection between MSC and ADCC therapy. MSCs not only act as a vehicle for BsAb secretion but also have differentiation capabilities to renew injured tissues. And the MSC-derived exosomes expressed MiRNA like MiR-15a and MiR-16, which target the BCL-2 gene and significantly inhibit the proliferation of Raji cells. Cumulatively, exosome from the nanoparticle-transferred MSCs plays a synergy role with anti-CD3/CD20 BsAb. Also, the exosome-based cell-free treatment will act as a new strategy for cell immunotherapy in the future.

## Supplementary data

Supplementary data are available at *Regenerative Biomaterials* online.

## Supplementary Material

rbac090_Supplementary_Data
