# Supplementary material for: Epsilon-caprolactone-modified polyethylenimine as a genetic vehicle for stem cell-based bispecific antibody and exosome synergistic therapy
Source: Regen Biomater. 2022 Nov 2;10:rbac090. doi: 10.1093/rb/rbac090 (PMC9847525; doi:10.1093/rb/rbac090)
Supplement: rbac090_Supplementary_Data [file rbac090_supplementary_data.docx]

**Epsilon-caprolactone Modified Polyethylenimine as a genetic vehicle for Stem Cell-based Bispecific Antibody and Exosome Synergistic Therapy**

Yan Tan^1^, Jiali Cai^2^, Zhiyong Wang^2^*

^1^Guangdong Key Laboratory for Biomedical Measurements and Ultrasound Imaging, School of Biomedical Engineering, Health Science Center, Shenzhen University, Shenzhen 518060, China

^2^Center for Functional Biomaterials, Key Laboratory for Polymeric Composite and Functional Materials of Ministry of Education, School of Materials Science and Engineering, Sun Yat-Sen University, Guangzhou 510275, China

^*^Corresponding address. E-mail: [wangzhiy3@mail.sysu.edu.cn](mailto:wangzhiy3@mail.sysu.edu.cn)

#### Supplementary figures.

Figure S1. The construction map of MC.CD20. The gene expression cassette includes CMV enhancer and promoter, anti-CD3/CD20 scFv gene sequence, flag tag, His tag, Kozak sequence, signal peptide, termination codon, and poly-A tail.


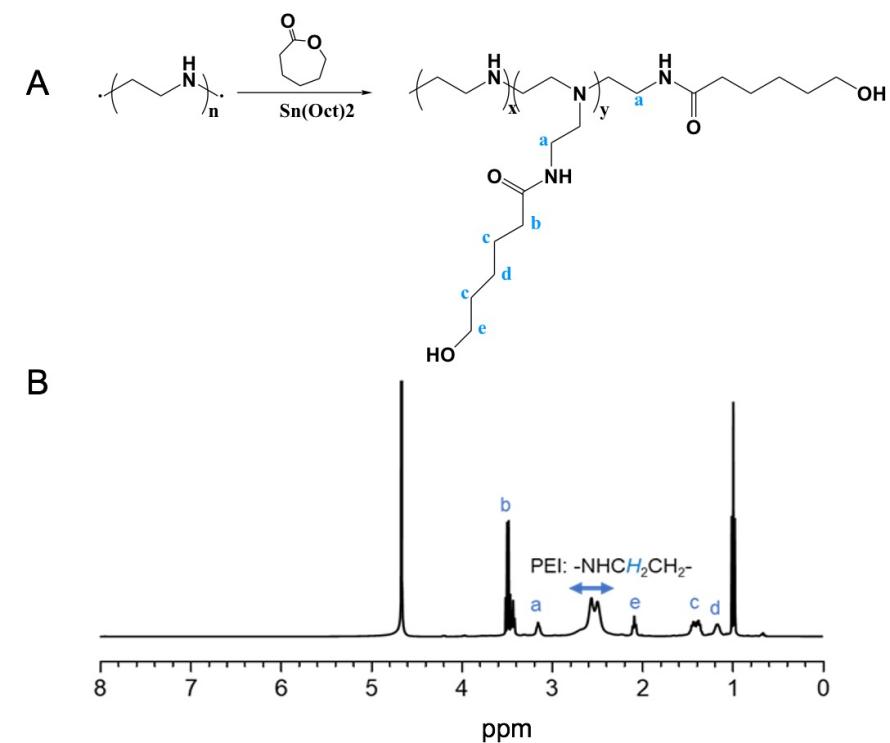


Figure S2. (A) Synthesis route of the PEI25K-CL; (B) ^1^H NMR of the PEI25K-CL.

characteristic peaks: 1.23 ppm (CL: -COCH_2_CH_2_*CH_2_*CH_2_CH_2_OH); 1.47 ppm(CL: -COCH_2_*CH_2_*CH_2_*CH_2_*CH_2_OH); 2.15 ppm (CL: -COCH_2_CH_2_CH_2_CH_2_*CH_2_*OH); 2.25-3.00ppm (PEI: -NHC*H*_2_CH_2_-，-NC*H*_2_CH_2_-); 3.20 ppm(PEI：-CH_2_CH_2_NHCO-，-CH_2_*CH_2_*NCO-); 3.50 ppm (CL: -CO*CH_2_*CH_2_CH_2_CH_2_CH_2_OH)


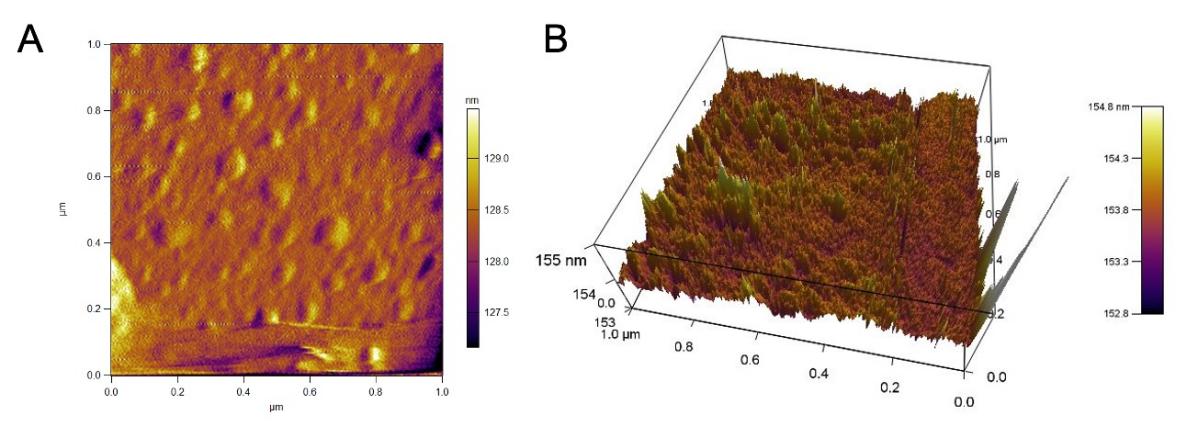


Figure S3. AFM imaging (tapping model)of exosome that derived from the PEI25K-CL/MCDNA transfected HucMSCs. (A-B) 2D and 3D morphology of the exosomes.


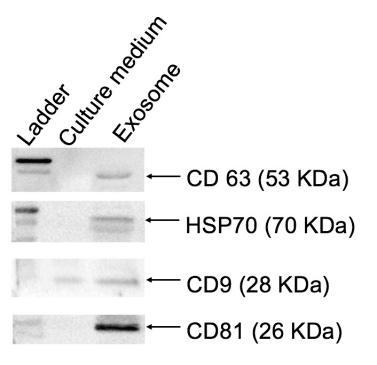


Figure S4. Western Blot experiment for exosome marker assay. After transfection by PEI25K-CL/MCDNA, the CD63, HSP70, CD9 and CD81in the culture medium and extracted exosomes were evaluated.


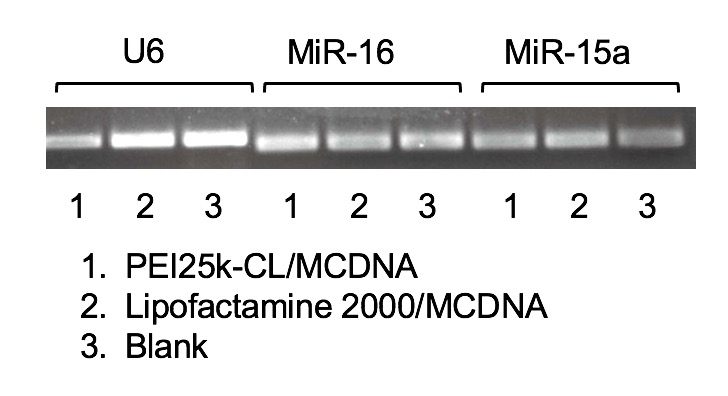


Figure S5. Electrophoresis gel experiments were performed to confirm that the PCR products expressed microRNAs.


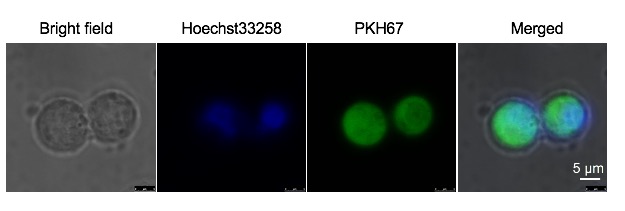


Figure S6. Confocal imaging of the Raji cells after incubated with PKH67 stained exosomes.

PKH 67 shows green and represents the cell internalized exosomes. Hoechst 33258 stains the nucleic of the Raji cell. The sale bar is 5 μm.
